# Acousto-optic modulators integrated on-chip

**DOI:** 10.1038/s41377-022-00928-z

**Published:** 2022-07-29

**Authors:** Jared Beller, Linbo Shao

**Affiliations:** Bradley Department of Electrical and Computer Engineering, Virginia Tech, Blacksburg, VA 24061 USA

**Keywords:** Integrated optics, Micro-optics

## Abstract

Acousto-optic devices that use radio frequency mechanical waves to manipulate light are critical components in many optical systems. Here, the researchers bring acousto-optic devices on-chip and make them more efficient for integrated photonic circuits.

Acousto-optics studies interactions between light and acoustic waves in a medium. The optical refractive index of a material changes in the presence of strain from acoustic waves. The effects of this, also called Brillouin scattering, were first predicted by Léon Brillouin in 1922^1^ and have been an active area of research since. With careful designs, acoustic waves can manipulate light in various ways, such as deflecting light into different spatial modes, modulating intensity, shifting frequency, and rotating polarization.

Acousto-optic modulators are widely used in many optical systems, including *Q*-switch lasers, ion traps, optical tweezers, spectrometers, and optical signal processors. Conventionally, acousto-optic devices use large bulk crystals with sizes of a few centimeters^2^. The acoustic waves are generated electrically from the piezoelectricity of a crystal and interact with light propagating through the crystal. Acousto-optic modulators offer outstanding performance including high extinction ratios, large carrier suppression, broad optical bandwidths, and fast responses, which are challenging to achieve using other approaches like electro-optics and thermo-optics.

Despite their outstanding performance, bulk acousto-optic devices are not compatible with emerging integrated optical platforms. These platforms contain multiple optical devices, all fabricated and interconnected on a single chip. Bringing acousto-optic devices on chip is not trivial. Innovations in device designs and material platforms are necessary to simultaneously confine both acoustic waves and light on chip and facilitate their interactions.

Recent efforts have been made to demonstrate integrated acousto-optic devices on chip. Much of this has focused on surface acoustic waves, which stay confined to the same layers as existing on-chip optical waveguides. Acoustic waves are generated with interdigital transducers placed on the surface of a thin-film piezoelectric material, such as zinc oxide^3^, gallium arsenide^4^, aluminum nitride^5,6^, or lithium niobate^7–11^. With recent advances in fabrication, lithium niobate has looked especially promising due to its high piezoelectric coefficients and low optical and acoustic propagation losses.

Unfortunately, the efficiency of the integrated acousto-optic modulators is far from their bulk counterparts, as the interaction lengths between light and acoustic waves are significantly shorter in integrated devices. For example, the deflection efficiency of an integrated acousto-optic deflector^7,8^ is limited to 3.5%—far from the over 90% efficiency of a bulk acousto-optic deflector.

In the article in *Light: Science & Applications*^12^, Wan et al. boost the efficiency of integrated acousto-optic modulators on the thin-film lithium niobate platform by heterogeneously integrating optical waveguides made of chalcogenide glass. Heterogeneous integration provides opportunities by bringing together materials with promising properties. While the strong piezoelectricity of lithium niobate enables an efficient generation of acoustic waves, the much larger acousto-optic coefficients of chalcogenide glass enhance acousto-optic interactions.

The authors demonstrated a heterogeneous acousto-optic Mach-Zehnder modulator in the push-pull configuration with the acoustic wave generating interdigital transducer between the two arms. The presented modulator has a low *V*_π_ of 2.5 V, leading to a low *V*_π_*L* of 0.03 V cm, where *V*_π_ is the voltage required to induce a π phase shift of light and *L* is the interaction length of the device. This marks a significant improvement in the efficiency of integrated acousto-optic devices. Compared to current integrated acousto-optic modulators with suspended structures, the presented acousto-optic modulator is fully supported by the substrate, is easier to fabrication, offers greater scalability for photonic integrated circuits, and is more robust in practical applications.

The thin-film lithium niobate^13^ is a rising-star platform for integrated photonics. Beyond high performance electro-optic modulators, necessary and high-performance optical components, including lasers^14^ and photodiodes^15^, are being heterogeneously integrated on the thin-film lithium niobate. Highly efficient acousto-optic devices, as presented by this work, will provide another powerful toolset to the lithium niobate platform. They could be used for developing microwave-to-optical converters, optical isolators, tunable filters and would be essential for the future of quantum photonics and microwave photonics.
